# Dynamics of major environmental disasters involving fire in the Brazilian Pantanal

**DOI:** 10.1038/s41598-023-49154-6

**Published:** 2023-12-07

**Authors:** Tatiane Deoti Pelissari, Paulo Eduardo Teodoro, Larissa Pereira Ribeiro Teodoro, Mendelson Lima, Dthenifer Cordeiro Santana, Fernando Saragosa Rossi, Daniel Henrique dos Santos, Renan De Almeida Silva, Thaís Lourençoni, Carlos Antonio da Silva Junior

**Affiliations:** 1 Department of Agronomy, State University of São Paulo (UNESP), Ilha Solteira, SP 15385-000 Brazil; 2 Federal University of Mato Grosso do Sul (UFMS), Chapadão do Sul, Mato Grosso do Sul 79560-000 Brazil; 3 State University of Mato Grosso (UNEMAT), Alta Floresta, Mato Grosso 78580000 Brazil; 4 Department of Geography, State University of Mato Grosso (UNEMAT), Sinop, Mato Grosso 78555000 Brazil

**Keywords:** Environmental sciences, Environmental social sciences

## Abstract

The 2020 environmental catastrophe in Pantanal has highlighted the fragility of environmental policies and practices for managing and fighting fires in this biome. Therefore, it is essential to know the causes and circumstances that potentiate these fires. This study aimed to: (I) assess the relationship between fire foci and carbon absorption (GPP), precipitation, and carbon dioxide (CO_2_) flux; (ii) analyze vegetation recovery using the differenced normalized burn ratio (ΔNBR) in Brazilian Pantanal between 2001 and 2022; and (iii) identify priority areas, where the highest intensities of fire foci have occurred, in order to guide public policies in Brazil to maintain local conservation. To this purpose, fire foci were detected using data from the MODIS MOD14/MYD14 algorithm, annual precipitation with CHIRPS (Climate Hazards Group InfraRed Precipitation with Station data), and CO_2_ flux using the MODIS/MODO9A1 product, and Gross Primary Production (GPP) with the MODIS/MOD17A2 product. The severity of the burned area was also assessed using the ΔNBR index and the risk areas were determined using the averages of these images. During the time series studied, a total of 300,127 fire foci were detected throughout the Pantanal, where 2020 had the highest number of foci and the lowest accumulated precipitation. The years with the highest precipitation were 2014 and 2018. The year 2018 was also the second year with the highest GPP value. The Pettit test showed a trend for 2008 and 2011 as the points of change in the CO_2_ flux and GPP variables. Principal component analysis clustered fire foci and precipitation on opposite sides, as well as GPP and CO_2_ flux, while ΔNBR clustered HS, MHS and MLS classes with the years 2020, 2019, 2002 and 2021. There was a high negative correlation between fire foci × rainfall and GPP × CO_2_ flux. The years with the largest areas of High severity (HS), Moderate-high severity (MHS) and Moderate-low severity (MLS) classes were 2020 and 2019, respectively. The most vulnerable areas for severe fires were the municipalities of Cáceres, Poconé, and Corumbá. The major fire catastrophe in 2020 is correlated with the low precipitation in 2019, the high precipitation in 2018, and the increased GPP, as well government policies unfavorable to the environment.

## Introduction

Pantanal is the largest floodplain on the planet and is home to a rich biodiversity, riverside communities, indigenous and quilombolas peoples. Its primary economic activity is cattle ranching, which can be considered sustainable by exploiting native pastures. In 2020, fires in Pantanal drew worldwide attention for their impact and environmental degradation, with millions of wild animals killed and a vast area affected. Approximately four million hectares of forest, savannah, and scrubland were burned^1–3^. However, there had already been a significant increase in fires in previous years^4^, as well as their frequency, with four major fires recorded in the last 14 years in the biome^5^.

Burning to clear pastures is common practice in region and, when applied improperly and combined with climatic conditions, changes in land use and poor conservation policies lead to an increasing occurrence and intensity of wildfires^6–8^. These recurrent fires promote a loss of biodiversity, replacement of native species by invasive ones, changes in ecological processes, impact on water quality, as well as changes in soil properties^9–13^. Additionally, the greenhouse gas (GHG) emissions resulting from these fires undermine the climate agreements signed by Brazil^4^.

Monitoring fires, vegetation behavior, and the extent of GHG emissions is essential for drawing up public policies that can at least minimize the effects of large fires such as the ones that occurred in 2020. Because of their large-scale detection ability, remote sensing (RS) techniques are used to map fire severity and progression, as well as to estimate carbon dioxide (CO_2_) emissions^14,15^. Furthermore, the impact of different levels of fires on the vegetation and ecological systems is poorly understood^16^, since the severity of fires is related to the changes, loss or consumption of organic matter under or on the soil^17^. Data on vegetation severity together with estimates of carbon dioxide (CO_2_) emissions from environmental fires are crucial for designing the recovery of ecosystems damaged by fire^18^.

Several spectral models have been used to monitor fires, the severity of burned areas, and CO_2_ emissions by orbital sensors. Vegetation indices based on optical data from passive sensors are used to assess the severity of fires^17^, mainly in the near-infrared wavelength. For example, the relativized burning rate (RBR)^19^, relative burning rate (RΔNBR)^20^, and differentiated normalized burn ratio (ΔNBR)^21^ can be used for this purpose.

These indices classify the degree of post-fire damage by using multispectral indices calculated as the ratio between the difference in near-infrared (NIR) and short-wave infrared (SWIR) band reflectance and the sum of NIR and SWIR band reflectance^22^. In short, this index assesses the changes in vegetation and soil reflectance caused by fires, since the reflectance of the SWIR spectral bands increases before and after the fire, while the NIR reflectance decreases^23^.

Evaluating changes in Gross Primary Production (GPP) before and after a fire can also be used as a way of assessing the severity of vegetation burning^11^, associating it to biomass^24^. After the GPP decreases when the vegetation is burned, it increases when the vegetation recovers and regrows^25^. Thus, fires and severe droughts have a significant impact on the vegetation and hence on the GPP^26^. Furthermore, in the burned area, there is an increased CO_2_ absorption as a result of vegetation regrowth and recovery of the post-fire ecosystem^27^. Fire impact on vegetation also depends on the moisture content of the soil organic matter and atmosphere, and the length and intensity of the dry season makes combustible materials more arid and favorable for burning^28^ Another important factor is that excess rainfall can increase vegetation biomass and hence increase fires in subsequent years of severe drought^29^, influencing the occurrence of fires in different ways, scales, and perspectives^30^.

Understanding the dynamics of events that can promote large fires in Pantanal is crucial for developing strategies to prevent these environmental disasters and their contribution to global warming. This information is essential for decision making, where planning includes the restoration of highly degraded areas and fire management in unburned areas, taking into account the time scale to prioritize actions in the short and long term^30^. This study aims to identify areas with a high risk of fire in order to establish priority municipalities for fire prevention and firefighting actions in the Pantanal biome. Therefore, this study aimed to evaluate (i) the relationship between fire foci and carbon absorption (GPP), precipitation, and carbon dioxide flux (CO_2_); (ii) analyzing vegetation recovery using differentiated normalized burn ratios (ΔNBR) in the Brazilian Pantanal between 2001 and 2022; and (iii) identify priority areas, where the highest intensities of fire foci have occurred, in order to guide public policies in Brazil to maintain local conservation.

## Results

### Exploratory data analysis

**Figure 1:**
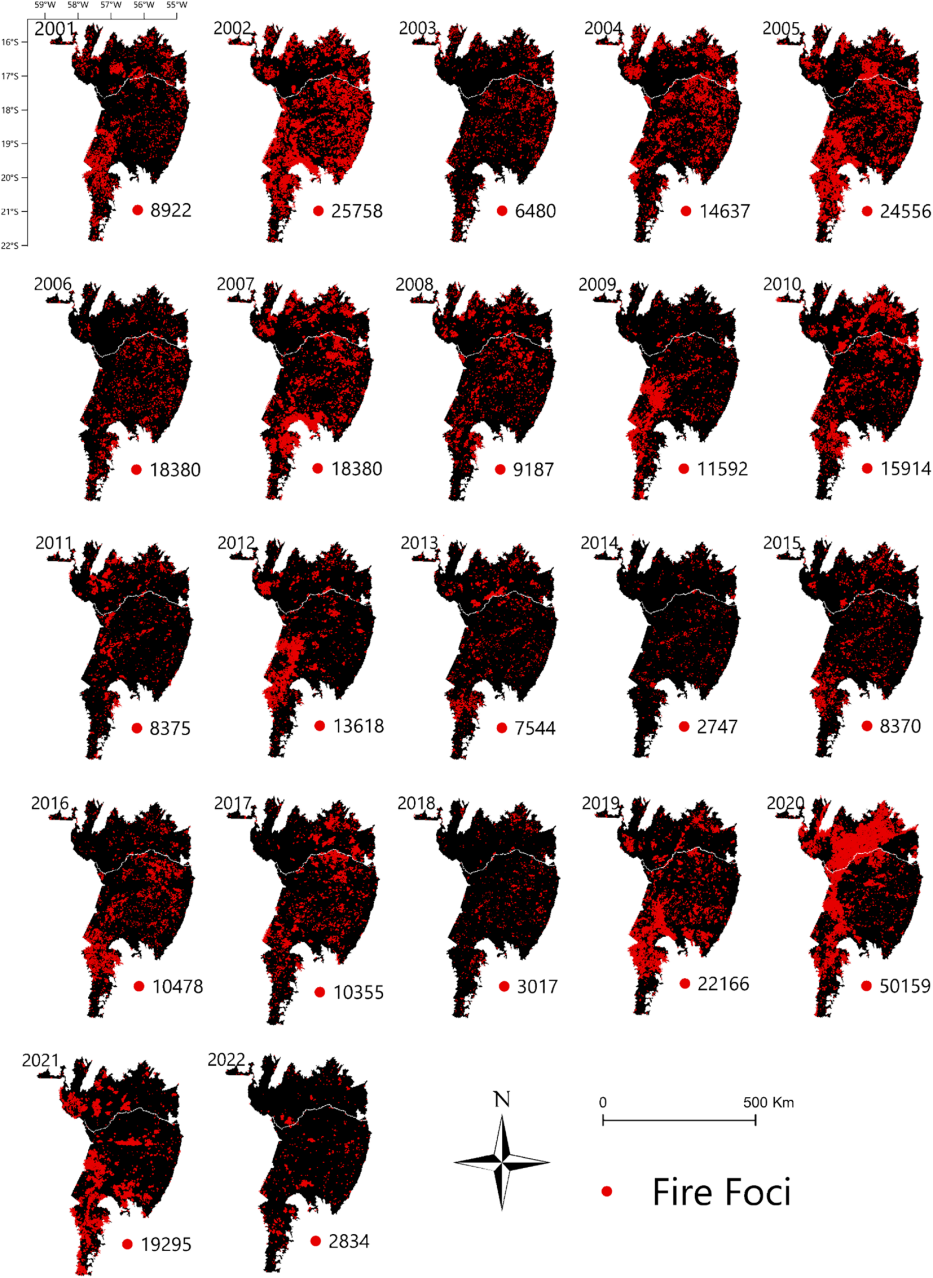
Fire foci monitoring in the Brazilian Pantanal biome using MODIS Thermal Anomalies/Fire Locations products, between 2001 and 2022. To prepare image was used the QGIS available through the Google Earth Engine platform (Google, https://earthengine.google.com/) through the dataset available at ee.Image (“MODIS/061/MOD14A1”).

From 2001 to 2022, 300,127 fire foci were detected throughout the Brazilian Pantanal biome (Fig. 1). The year with the highest absolute number of fire foci was 2020, with 50,159 outbreaks. This year also had the highest mean for the time series (4106.08) along with 2002 (2150.58) (Supplementary Table 1). The most affected State was Mato Grosso do Sul (MS), with 201,542 foci, which accounted for 67% of the total foci. When analyzing the number of fire foci per km^2^, Mato Grosso do Sul also had the highest values, 0.021 per km^2^, while Mato Grosso (MT) had 0.018 per km^2^ (Supplementary Table 2).

**Figure 2:**
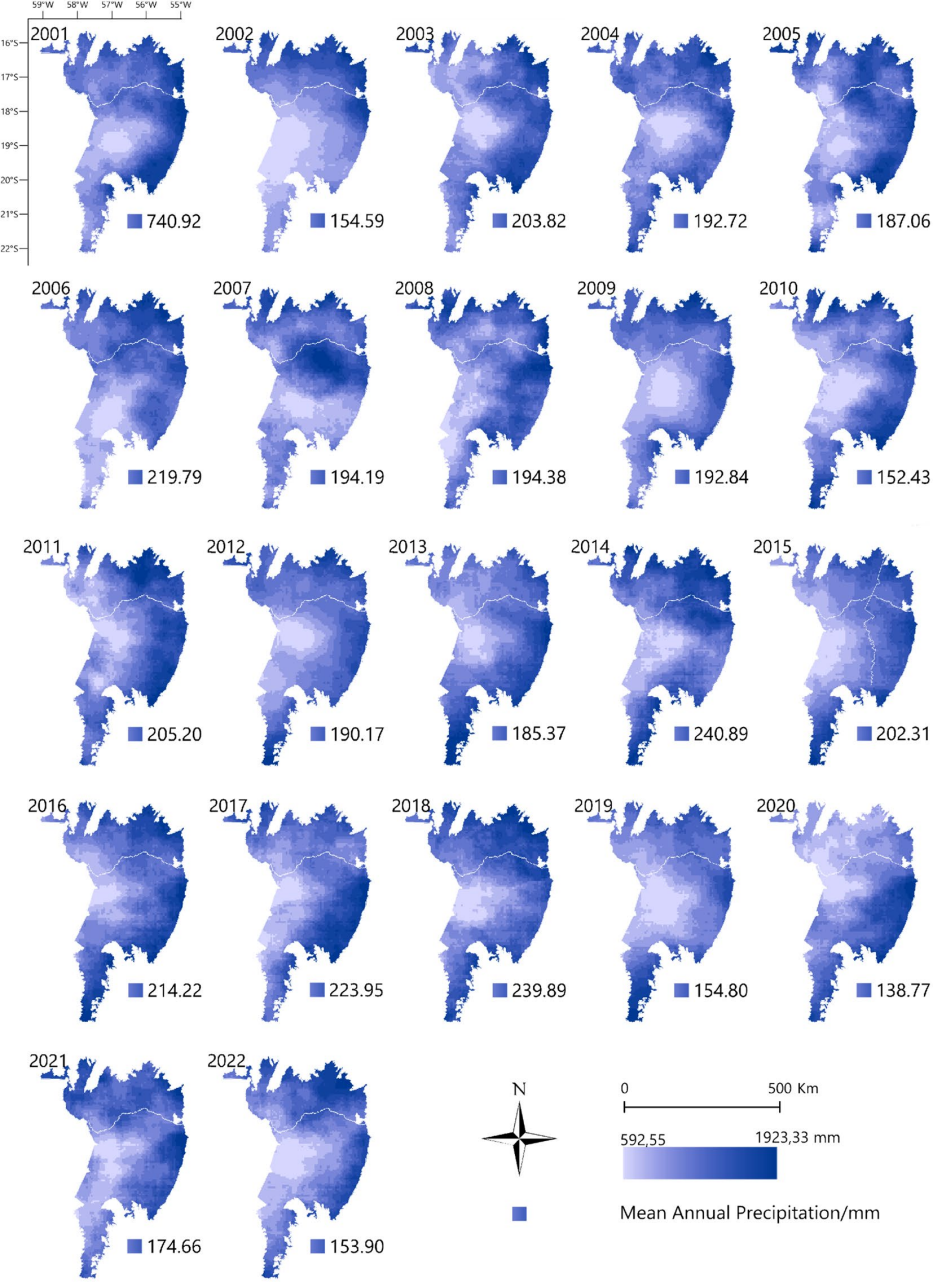
Monitoring annual accumulated precipitation in mm, via CHIRPS data, in the Brazilian Pantanal biome between 2001 and 2022. To prepare image was used the QGIS available through the Google Earth Engine platform (Google, https://earthengine.google.com/) through the dataset available at ee.Image (“UCSB-CHG/CHIRPS/DAILY”).

Figure 2 shows a 22-year time series (2001 to 2022) of precipitation monitoring via CHIRPS data across the Pantanal biome. There was high data variability throughout the Pantanal biome. The municipalities of Corumbá and Ladário showed a pattern of lower rainfall during the years of the time series.

The years with the highest accumulated and average rainfall were 2014 (2890.72 mm accumulated and 240.89 mm average) and 2018 (2878.7 mm accumulated and 239.89 mm average). The years with the lowest accumulated values were 2020 (1665.25 mm), mainly in the northern region of the biome, followed by 2010 (1829.13 mm). The difference in accumulated value between the year with the highest (2014) and the year with the lowest rainfall (2020) was 1225.47 mm (Supplementary Table 3).

**Figure 3:**
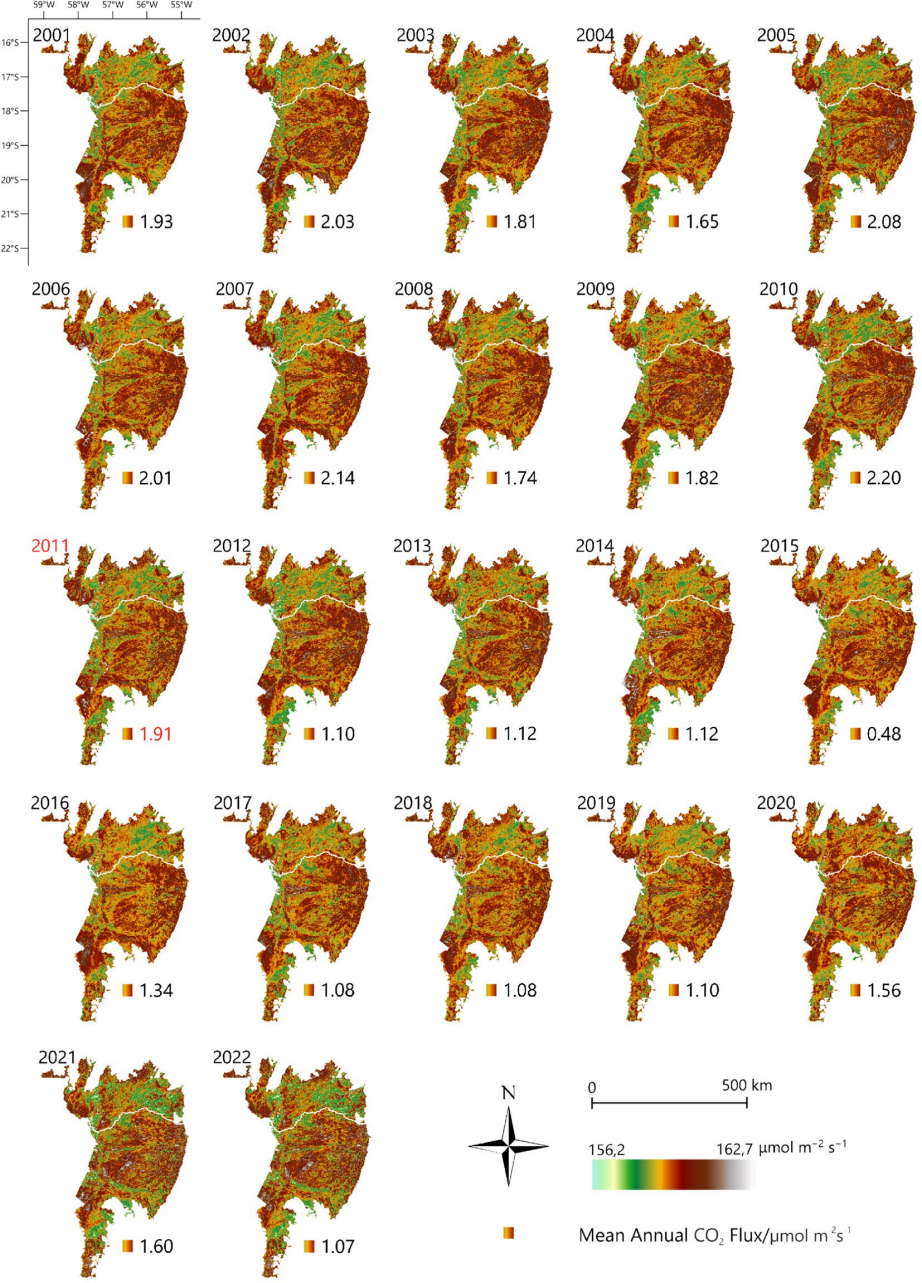
Monitoring CO_2_ flux using MODIS/MOD09A1 products in the Brazilian Pantanal biome between 2001 and 2022. The year 2011 was indicated by the Mann–Kendall test as a probable year of change. To prepare image was used the QGIS with data available through the Google Earth Engine platform (Google, https://earthengine.google.com/) through the dataset available at ee.Image (“MODIS/061/MOD09A1”).

Figure 3 shows the dynamics of the CO_2_ flux model over the entire time series (2001 to 2022). The municipalities in which the image shows the highest intensification of CO_2_ emissions were Corumbá, mainly in the southwestern region, and the municipalities of Sonora, Coxim, and Rio Verde de Mato Grosso in the eastern region of the biome. The years with the highest mean CO_2_ emissions were 2005 (162.6 μmol m^−2^ s^−1^), 2015 (112.4 μmol m^−2^ s^−1^), and 2020 (156.2 μmol m^−2^ s^−1^) (Supplementary Table 4).

**Figure 4:**
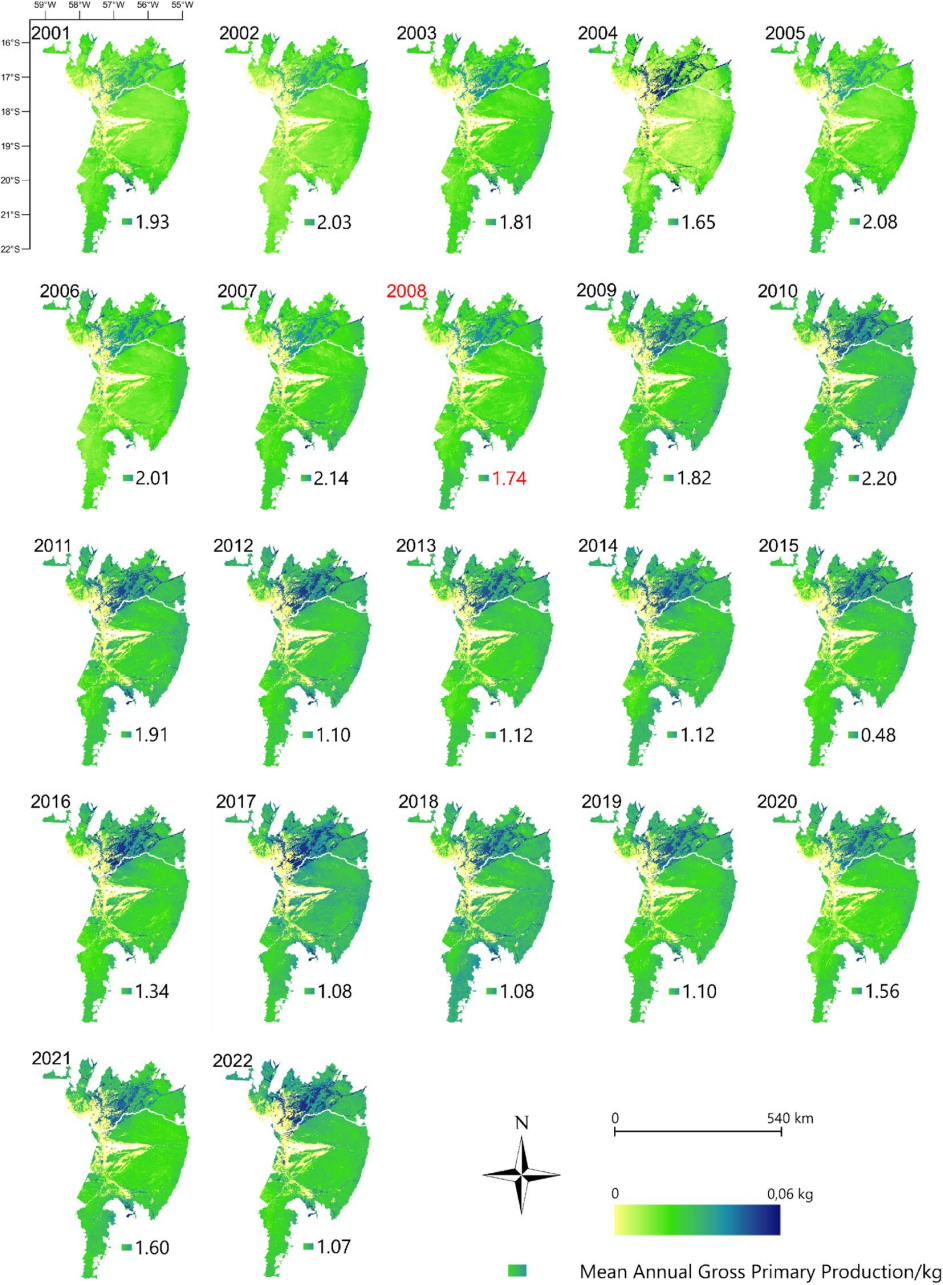
Gross primary production (GPP) via MODIS/MOD17A2 data in the Brazilian Pantanal between 2001 and 2022. The year 2008 was indicated by the Mann–Kendall test as a probable year of change. To prepare image was used the QGIS with data available through the Google Earth Engine platform (Google, https://earthengine.google.com/) through the dataset available at ee.Image (“MODIS/061/MOD17A2H”).

Figure 4 shows the mean gross primary production (GPP) over the years 2001 to 2022 throughout the Pantanal biome. The highest GPP was observed in the northern Pantanal biome, mainly in the municipalities of Poconé and Barão de Melgaço. The lowest GPP was evident in the region bordering the Brazilian Pantanal with Bolivia, in the western region. The highest annual mean GPP values, considering the entire Pantanal biome, were recorded in 2018 (0.0179 kg) and 2017 (0.019) (Supplementary Table 5).

### Trend analysis

Mann-Kendal test applied to the variables evaluated (GPP, precipitation, fire foci, and CO_2_ flux) detected a significant increase in trend for the GPP and CO_2_ flux variables for both States. Pettitt test then identified 2008 as the likely point of change in the time series for the GPP variable, and 2011 for the CO_2_ flux variable. No change points were identified for the precipitation and fire foci variables (Table 1).

**Table 1:** P-value of the Mann–Kendall and Pettit tests at 5% significance for the time series from 2001 to 2022 applied to the variables GPP, rainfall, CO_2_ flux, and fire foci in the States of Mato Grosso and Mato Grosso do Sul.

Test	MT				MS			
	GPP	Precipitation	CO_2_ Flux	Fire foci	GPP	Rainfall	CO_2_ Flux	Fire foci
Mann–Kendall	0.00	0.57	0.02	0.34	0.00	0.78	0.00	0.74
Petitt	0.00	0.25	0.00	0.67	0.00	0.29	0.00	1.0
Point of change	2008	–	2011		2008	–	2011	–

**Figure 5:**
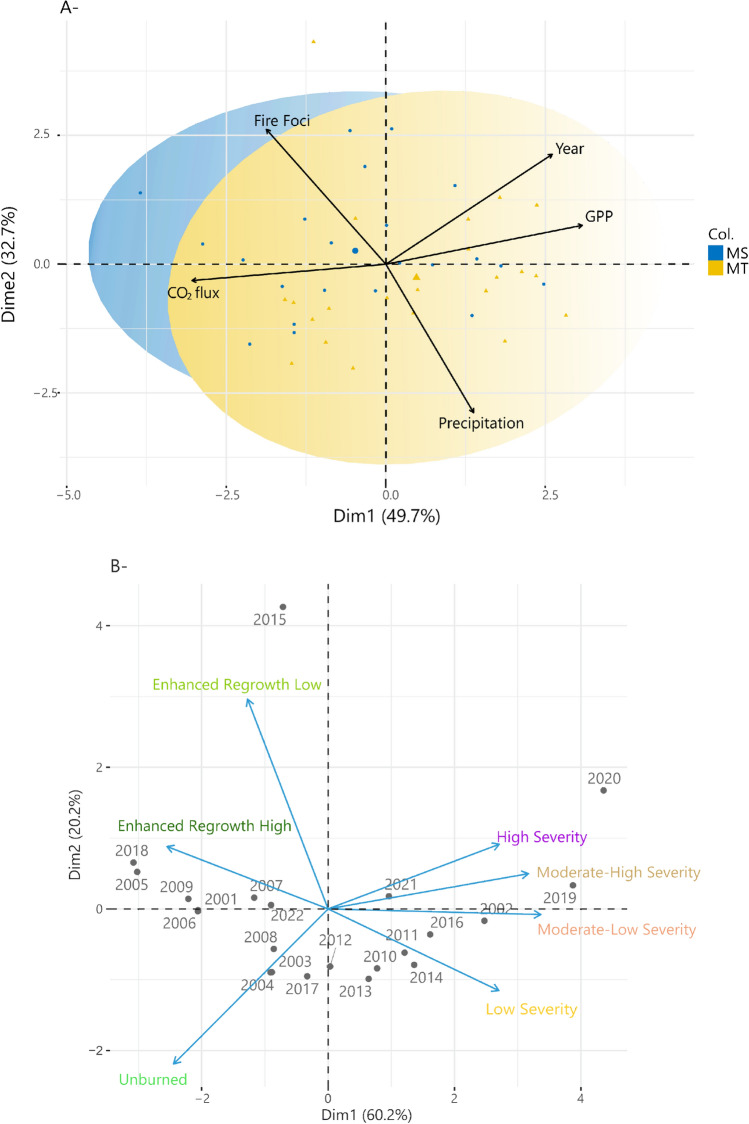
A. Principal component analysis for the years 2001–2022 of the variables GPP, precipitation, CO_2_ flux, and fire foci in the States of Mato Grosso and Mato Grosso do Sul. B. Principal component analysis of the ∆NBR index classification for the entire Brazilian Pantanal between 2001 a 2022. Package used of R to create the figure was “ggfortify” (v0.4.16, https://cran.r-project.org/web/packages/ggfortify/index.html).

According to the PCA (Fig. 5) applied to two sets of data across the Brazilian Pantanal biome, the sum of the first components in both analyses explains more than 70% of the variation in the data, indicating that both analyses were considered statistically appropriate^31^. The first graph (Fig. 5A) shows the variables fire outbreaks, precipitation, GPP, and yearly CO_2_ flux for the two States evaluated. Year and GPP variables were vectors opposite the CO_2_ flux vector, showing that when there is an increase in GPP there is a decrease in CO_2_ emissions. Fire foci distributed in the quadrant opposite the rainfall support the fact that when there was a higher incidence of rainfall there was a lower number of fire foci.

Figure 5B shows the data for the ∆NBR index classes (ERH, ERL, UM, LS, MLS and HS). The ERH and ERL classes were grouped together for the years 2005, 2007, 2009, 2015, and 2018. The HS and MHS classes were close to the years 2020, 2019, and 2021. This grouping shows that in these years, the fires with the greatest extensions and severity occurred during the time series (Fig. 5).

**Figure 6:**
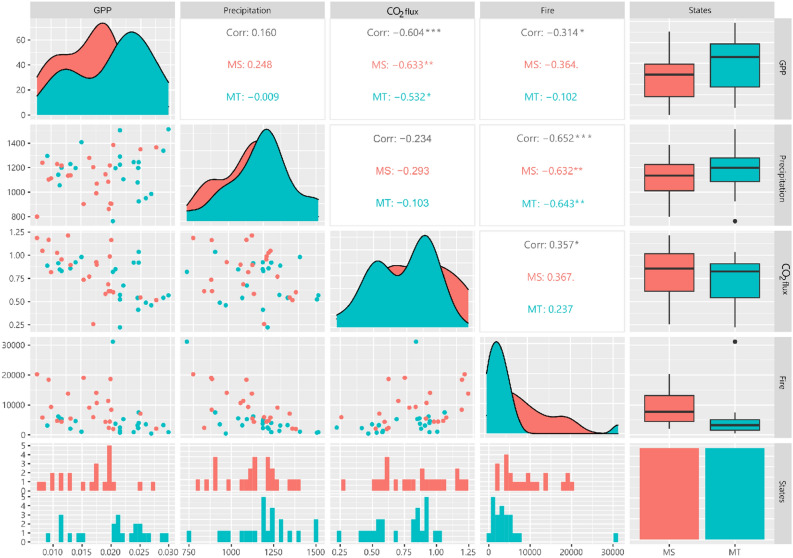
Pearson's correlation analysis for the variables rainfall, CO_2_ flux, GPP and fire foci within the States of Mato Grosso and Mato Grosso do Sul, in the Pantanal biome. Package used of R to create the figure was “GGally” (v2.1.2, https://cran.r-project.org/web/packages/GGally/index.html).

After grouping the data between the States, a Pearson correlation analysis was carried out between the variables GPP, precipitation, CO_2_ flux, and fire foci in the States of Mato Grosso and Mato Grosso do Sul. Figure 6 shows that there was a high negative correlation between the GPP variables and the CO_2_ flux, both for the entire Pantanal (− 0.604) and States of Mato Grosso (− 0.532) and Mato Grosso do Sul (− 0.633). A high negative correlation can also be observed between fire foci and precipitation for the entire biome (− 0.652), as well as for the States of MT (− 0.643) and MS (− 0.632).

Table 2 shows the results of the trend test applied to the time series of each class considered. No trend (p-value ≤ 0.05) was found for the ΔNBR classes (ERH, ERL, UN, LS, MLS, MHS, HS). Consequently, the Pettitt test did not identify any change points.

**Table 2:** P-values of the Mann–Kendall and Pettit tests of the time series of ΔNBR class variables (ERH, ERL, UN, LS, MLS, MHS, HS) in the Brazilian Pantanal biome.

ΔNBR Classes							
Test	ERH	ERL	UN	LS	MLS	SHS	HS
Mann–Kendall	− 0.12	− 0.15	− 0.42	0.20	0.17	0.39	0.36
Petitt	0.25	0.37	0.81	0.13	0.13	0.13	0.15
Ponto de mudança	–	–	–	–	–	–	–

**Figure 7:**
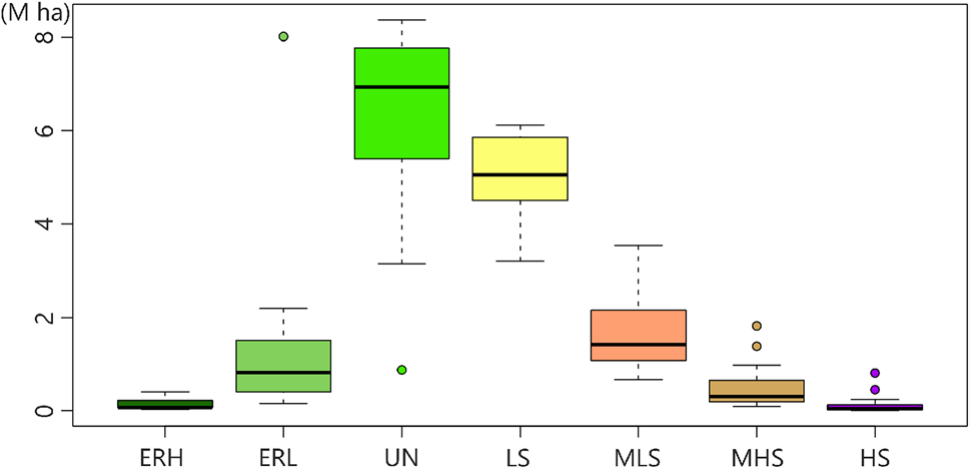
Boxplot for the ΔNBR index classes: Enhanced regrowth high, Enhanced regrowth low, Unburned, Low severity, Moderate-low severity, Moderate-high severity, and High severity for the entire Pantanal biome. Package used of R to create the line and boxplot graphs were “ggplot2” (v3.2.1, https://cran.r-project.org/web/packages/ggplot2/index.html).

Boxplot analysis applied to the ΔNBR index classes in the Pantanal biome indicates that the classes with the highest mean values were UN and LS (Fig. 7). MHS and HS were the classes with the highest number of outliers, or data with the most discrepant values. The ERL class had the outlier furthest from the median.

**Figure 8:**
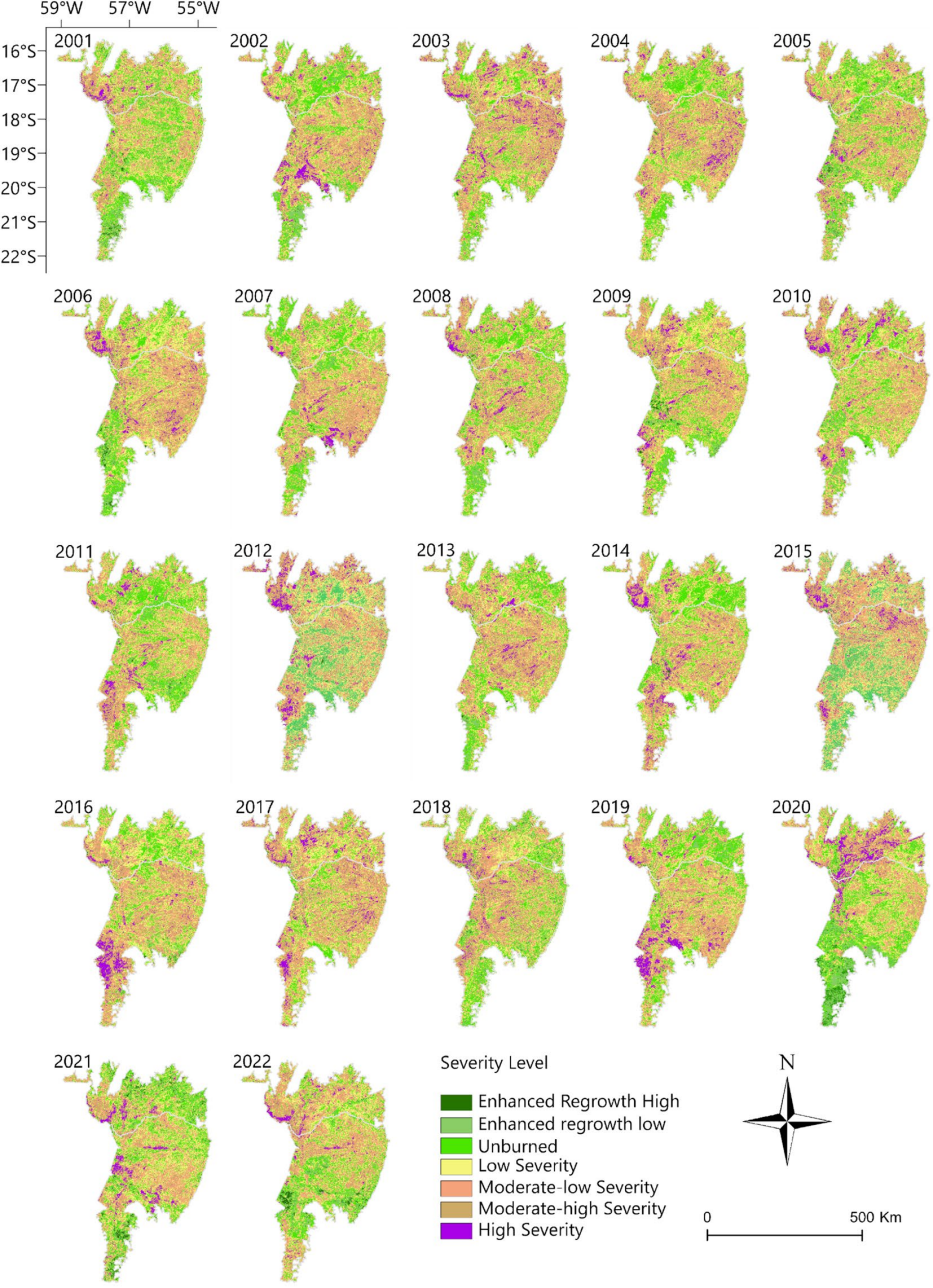
Classification of the ΔNBR index, using the product for the Brazilian Pantanal biome, between the years 2001–2022. To prepare image was used the QGIS available through the Google Earth Engine platform (Google, https://earthengine.google.com/) through the dataset available at ee.Image (“MODIS/061/MOD14A1”).

Figure 8 shows the areas of the Pantanal biome that were classified with the ΔNBR index during the study period (2001 to 2022). The ∆NBR shows that there have been fires over the years in different parts of the Pantanal. However, the MHS and HS classes were very evident in 2020, 2019, and 2002, and in 2020 there was the greatest detection of the High Severity class in the entire time series, highlighting the place where there was the greatest detection of GPP (see Fig. 4), for a large portion of the study period. The southwestern region of the municipality of Corumbá had areas classified as MLS, MHS, and HS in various years, with this being most evident in 2002, 2011, 2014, 2016, and 2019. In the municipality of Cáceres, in the region bordering Bolivia, areas classified as HS were also identified. In 2020, the largest area classified as HS was detected, with 811,558 ha, followed by 2019 with 455,988 ha, the highest values identified throughout the time series (Supplementary Table 6). The years 2020 (6,178,664 ha) and 2019 (5,308,235 ha) were also the ones with the highest number of areas classified with the sum of the MLS, MSH and HS classes.

**Figure 9:**
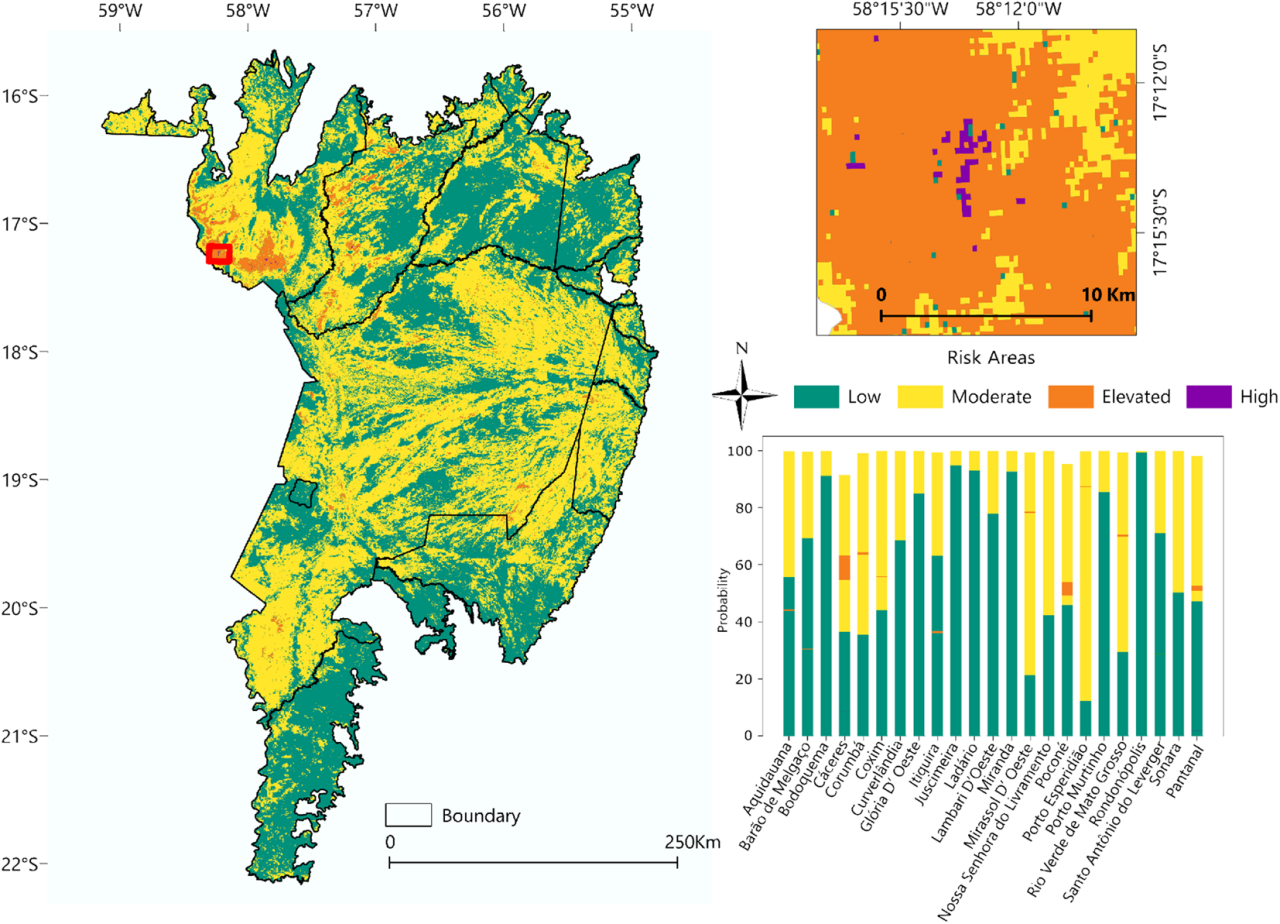
Risk areas for the entire Brazilian Pantanal biome, mapped using the means of the MLS, MHS, and HS classes of the ∆NBR index. To prepare image was used the QGIS available through the Google Earth Engine platform (Google, https://earthengine.google.com/) through the dataset available at ee.Image (“MODIS/061/MOD14A1”).

Figure 9 shows the probability of fires occurring in each municipality of the biome, as well as for the entire Pantanal biome. The occurrence of areas with moderate risk predominated in a large part of the biome, mainly in the municipalities of Corumbá, Sonora, Rio Verde de Mato Grosso, Cáceres, and Poconé. The areas mapped as elevated risk were detected mainly in the municipalities of Cáceres and Poconé and in Corumbá. The areas mapped as high risk, where there is a high probability of fires, occurred in the municipalities of Cáceres and Poconé. The municipalities classified as having a high or very high risk of fires were Cáceres, Poconé, and Corumbá.

## Discussion

In 2019 and 2020 the Pantanal faced one of its worst droughts, with decreasing rainfall levels and high temperatures and high heatwaves^32–34^. As a result, there was an increase in evapotranspiration rates, leading to a loss of moisture in the soil and vegetation^35–37^. Likewise, river levels have dropped, leaving organic matter available to burn slowly for weeks, which has increased the severity of the fire^38^. In addition to these factors, 2018 had the second highest rainfall, favoring the accumulation of organic matter^29^, which was subsequently dried out by the drought of 2019 and 2020 and intensified the availability of fuels for the catastrophic fires of 2020^34,39^. All these factors together have resulted in 11,486,899 ha of burned areas.

The expansion and severity of the burned area were accentuated by the slope of the region, cleared areas, the availability of fuel, and the induction of drought in pastures where practices were adopted to prevent flooding, such as blocking water flow channels. These factors allowed the fire to spread easily and quickly, while some of them made it difficult to control and fight the fire^13,40^.

Fire control was still difficult to carry out in remote regions and locations where underground fires occurred^2^. In addition, during the COVID-19 pandemic, which peaked in 2020, indigenous firefighters, approximately two-thirds of the firefighters working in the State of Mato Grosso do Sul, were unable to work during the pandemic. Firefighters have also had to follow safety protocols with social distancing, causing work overload^41^ and difficulty in fighting the fire.

This set of climatic, environmental, and human factors has been further intensified by unfavorable federal government decisions regarding environmental law enforcement procedures for illegal burning activities and shortcut of budget for fire combat and prevention, as reported by^42,43^. As well as the extinction of important agendas, such as the Climate Change Secretariat^44^.

Mann–Kendall test applied to the variables precipitation, fire foci, GPP, and CO_2_ flux showed an upward trend for GPP and CO_2_ flux. The year 2008 was pointed out as the trend point for a significant change in GPP, given that in the previous years, 2007 (21,832,001 cattle) and 2008 (22,365,219 cattle), the State of Mato Grosso do Sul recorded a decrease of up to 12% in its cattle herd, compared to the peak year 2003 (2,498,3821 cattle)^45^. As the number of cattle decreases, there is less grass intake, consequently increasing the GPP of the pastures^24,46^, as well as reducing the demand for new areas for livestock farming and preserving native vegetation. ^47^ point out that the high density of cattle and changes in land use influence burning patterns, and also observed a 30% reduction in the photosynthetic productivity of vegetation. In 2011, a significant trend was detected for the CO_2_ flux, which was affected by strong and moderate La Niña events^48^. During ENSO (*El Niño Southern Oscillation*) events, fire flows are one of the main factors in carbon-based gas emissions and growth rates^49,50^. Thus, the influence of climatic phenomena on the dynamics of fires in the Pantanal biome is recognized^51^.

The areas identified here as having a high probability of environmental fires are mostly in the municipalities of Cáceres and Poconé, in the State of MT, and Corumbá, in the State of MS. Corumbá is the municipality with the largest area in Mato Grosso do Sul (64,432.450 ha), has the second largest GDP in the biome and the third largest economy in Mato Grosso do Sul, with one of the largest populations (96,268 inhabitants) in the Pantanal^52^. Cáceres is considered the main Pantanal municipality in the State of Mato Grosso and has the largest cattle herd in the State, with 1,161,605 cattle, as well as the second largest number of farm properties dedicated to livestock farming and the third largest GDP of the Pantanal municipalities (IBGE, 2020; IBGE, 2021). The municipality of Poconé is the gateway to the Pantanal in MT, with several tourist establishments around the Transpantaneira Highway (Zelito Dorileo Highway). Besides tourism, other economic activities stand out, such as livestock farming, with 526,275 head of cattle, mining, and agriculture^45,53^ According to the Platform^54^ in the last four harvest years, there has been a 150% growth in soybean areas in Cáceres and an 86.19% growth in Poconé, and in the 2022/2023 harvest year, a cultivated area of 16,804 ha in Cáceres and 10,602 ha in Poconé was identified. Along with soybean, sugarcane also stands out as one of the biome's major crops, which was allowed to be planted in Pantanal in 2019 by then-president Jair Messias Bolsonaro, under protest from the scientific community due to the risk of fires occurring^34,55^.

Given the above, we can expect a recurrence of fires in these municipalities if there isn’t appropriate planning aimed at mitigating fires and restoring the fire regime in the Pantanal biome in overall terms. For better efficiency in fire planning and management, it is necessary to monitor active fires, detect fire foci, and especially map the potential danger^56^, because mapping risk areas makes it possible to localise risk elements and the communities most prone to fires, making it essential for land managers, firefighters and fire brigades to plan emergency measures to deal with fires in real-time^57^. This is an important factor because, as a rule, investments in fire management and control tend to contain fires^58^.

Although in July 2020 the national government decreed a fire moratorium, which banned the use of fire for four months during the dry season, especially in the Amazon and Pantanal biomes, it did not guarantee that in 2020 the Pantanal biome would face its worst fire in decades^59^. Lack of consolidated environmental legislation covering the entire biome^60^, the low budgets of agencies such as IBAMA and the lack of inspection and monitoring due to the limited number of inspectors reinforce the concept of impunity and responsibility of offenders^13^.

The Pantanal needs its own policy plan that covers its entire territory and together with the States of Mato Grosso and Mato Grosso do Sul. Therefore, in addition to climatic factors, the legislative instruments should consider issues such as: controlling and reducing fuel loads, providing structures, facilities, and technology to help access hard-to-reach native areas, and improving water access and technology for fighting underground fires. Furthermore, changes in the landscape (altered and/or abandoned areas with high fuel loads) must be considered, as well as the social and economic issues of the Pantanal population^61^.

Aiming to reduce and prevent the occurrence of fires in the Pantanal biome, federal government agencies established the Action Plan for Integrated Fire Management in the Pantanal Biome (MIF), which seeks to associate firefighting with the needs of traditional fire use and the ecological and socio-economic aspects of the biome^62^. However, to establish Integrated Fire Management (IFM) in these areas, joint investment and action by state governments is essential, since they are in charge of most environmental regulation and inspection on private land^43^.

Another important issue is reducing the use of fire as an agricultural practice. Besides the ban, it is necessary for these farmers, especially the small and traditional ones, to have access to the technologies available to replace these practices. To this end, the government needs to make financial resources available for financing and rural credit for expenditure on goods and services to replace the use of fire, with payment terms that small producers and family farmers can afford.

It is also necessary to invest in the creation of fire brigades, equipment, and the training of a specialized workforce to combat and deal with fires. In this regard, mapping risk areas is vital to allocate sites for fire brigades, giving priority to those with the highest risks, such as Poconé, Cáceres, and Corumbá. Furthermore, the adoption of practices such as preventive controlled burning, already provided by the New Forest Code (Law No. 12.651/2012), carried out by firefighters or competent bodies before the dry season^63^ in fire-prone areas, is crucial for reducing the fuel and hence avoiding major disasters in the future.

Catastrophic fires in 2020 are directly correlated with the climatic factors precipitation^34,39^, GPP, and CO_2_ flux. The high rainfall accumulated in 2018 led to an increased GPP^29^, which was subsequently transformed into dry matter with the drought of 2019, which fueled the fires of 2020 and consequently increased CO_2_ emissions. The ∆NBR index classification identified severely burned areas in all years across the Pantanal biome, with the highest proportion in 2019 and 2020, mainly in the regions of Cáceres, Poconé, both in the State of MT, and Corumbá in MS. In some cases, the ∆nbr classification has some limitations due to the distinction between vegetation or non-fire-related images with variations in water bodies and continuous changes in vegetation^64,65^. Moreover, it is essential to analyse the temporal variations of carbon emissions and fires in order to design effective strategies to mitigate both fires and carbon emissions^66^.

This study shows that it is possible to predict new catastrophes. In a scenario for drawing up an environmental plan to mitigate fires in the Pantanal biome, it is necessary to define the following issues: (i) establishing priority areas for the implementation of fire-fighting infrastructure; (ii) mapping areas with high potential for organic matter in order to monitor these areas; (iii) investment in funding, technology, and approaches to replace fire as an agricultural practice. Changes in the biome landscape, coupled with climate change, demand greater attention and sensitivity from Brazilian governments, as well as new approaches to fighting fires to guarantee the biome’s environmental safety.

## Methods

### Study area

**Figure 10:**
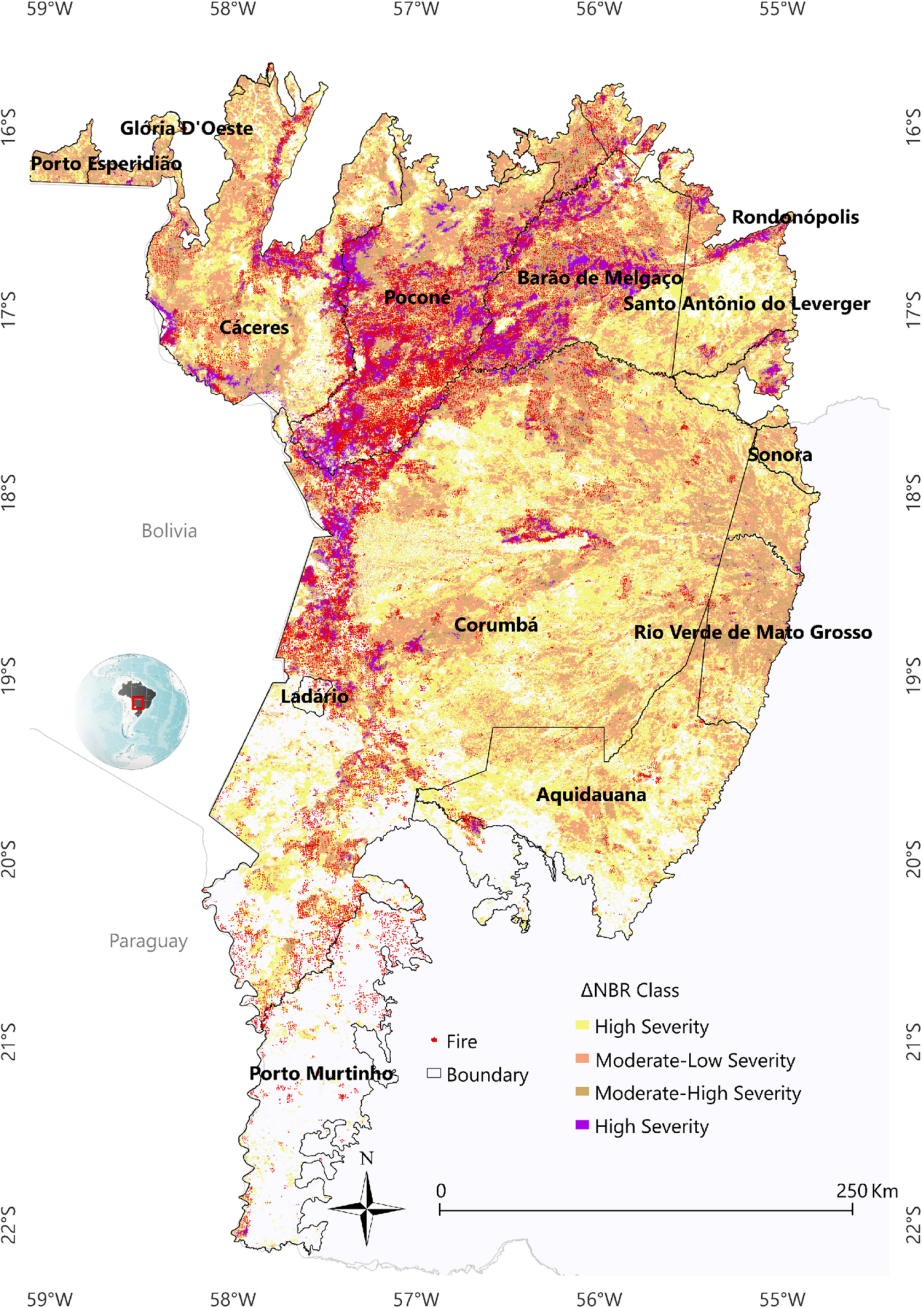
Study area comprising the Pantanal biome, delimited by the States of Mato Grosso and Mato Grosso do Sul. The figure shows the fire foci using the MODIS Thermal Anomalies/Fire Locations products, and the ΔNBR index classes using the MODIS/MOD13Q1 products. To prepare image was used the QGIS available through the Google Earth Engine platform (Google, https://earthengine.google.com/) through the dataset available at ee.Image (“MODIS/061/MOD14A1”).

The study area comprises the Brazilian Pantanal biome (Fig. 10), located in the central region of the South American continent, in the Alto Paraguay river basin, between the geographical coordinates 22° 0′ 0" S and 55° 0′ 0'' W, covering an area of 138,183 km^2^ across the States of Mato Grosso do Sul (65%) and Mato Grosso (35%)^67^. According to the Köppen-Geiger classification, the Pantanal's climate is Aw, a tropical climate with a rainy season in summer and a dry winter^68^. Annual rainfall is highest in the plateau areas, north-northeast (2000 mm) and south (1800 mm), and lowest in the central Pantanal (900 mm)^69^.

### Fire foci data

Fire foci data were calculated using the MODIS MCD14DL sensor (TERRA/AQUA). We used collection 6—Near real-time (NRT) MODIS thermal anomalies/fire locations- processed by NASA's Land, Atmosphere. The thermal anomalies/active fire represent the 1 km pixel center that is flagged by the MODIS MOD14/MYD14 algorithm^70^.

The final database consisted of a database from 2001 to 2022 containing fire_archive_M6 = Thermal Anomalies/Fire Locations of MODIS standard quality processed by the University of Maryland with a three-month delay and distributed by the FIRMS platform. The time period for fire foci was defined as from the first day of the year to the last day of each year (January 1st to December 31st), which is the time series for the entire area of the Brazilian Pantanal biome.

### Precipitation frequency analysis using CHIRPS data

The Google Earth Engine platform was used to extract the precipitation values from the CHIRPS (Climate Hazards Group InfraRed Precipitation with Station data) dataset using JavaScript code programming. The dataset provides daily precipitation values in mm/day with pixels of 5.566 km^71^. The images were cropped for the study area and exported in ".tif" format for processing in the software, using Qgis 3.28 software, and correlated with other data. Furthermore, the sum of monthly and annual precipitation from 2001 to 2022 was extracted for the entire Brazilian Pantanal biome.

### Estimating carbon sequestration efficiency

#### ***CO***_***2***_*** flux***

Data from the MODIS sensor orbited by the TERRA and AQUA satellites was used to monitor CO_2_ flux, using orbitally corrected reflectance images with a maximum of 20% clouds in each pixel, using the Google Earth Engine platform, by accessing the MODO9A1 product to obtain the entire time series, using the same methodology used by^72^. The dynamics of carbon sequestration in the Pantanal biome were evaluated over the years of the time series. For this purpose, the CO_2_ flux index model was used^73,74^. The purpose of this model is to measure the efficiency of the carbon sequestration process by vegetation, i.e., the photosynthetic rate during the photosynthesis process. The Photosynthetic Vegetation Index—PRI will be calculated (Eq. 1)^75^. The green and blue spectral bands were used to produce this index. The PRI estimates the carotenoid pigments in the foliage. These pigments, in turn, indicate the rate of carbon dioxide storage in the leaves^74^

1 $$PRI= \frac{A-Ve}{A+Ve}$$

*A*, blue range reflectance; *Ve*, green range reflectance.

However, the PRI results need to be rescaled, resulting in positive values. For this purpose, it is necessary to generate the sPRI (Eq. 2)^76^.

2 $$sPRI= \frac{(PRI+1)}{2}$$

Thus, the CO_2_ flux index model (µmol m^−2^ s^−1^) will be the result of the multiplication between NDVI and sPRI, in which there is a relationship between the PRI index, which indicates light-use efficiency in photosynthesis, with NDVI, which indicates the vigor of photosynthetically active vegetation, in which it may be able to capture absorptions from carbon sequestration. Thus, the best correlation is given in Eq. (3)^73,74,77^.

3 $${CO}_{2}FLUX=13.63-(66.207\cdot \left(NDVI\cdot sPRI\right)$$

#### Gross primary production (GPP)

MOD17A2 Gross Primary Production product is a cumulative composite of GPP values based on the efficient radiation use by vegetation (ε). By this logic, primary production is related to the photosynthetically active radiation absorbed (Eq. 4).

4 $$GPP=\upvarepsilon \cdot \mathrm{PA}\cdot \mathrm{FPAR}$$

A major challenge in using these models is obtaining the light-use efficiency “ε” over large areas due to its dependence on environmental factors and the vegetation itself^78^. One of the solutions consists of relating “ε” according to its maximum value (εmax) by adding more environmental contributions synthesized by the minimum air temperature (Tminscalar) and the state of water in the vegetation (Eq. 5).

5 $$\varepsilon ={\varepsilon }_{max}\cdot {T}_{minscalar}\cdot {VPD}_{scalar}$$

In this study, MODIS GPP version 6.1 was used together with the Google Earth Engine platform. Pixel values with reference to the digital numbers of the MODIS image were converted into biophysical values (kg C m^−2^) at scale 0.0001^79^ GPP values were transformed to 8-day average values measured in kg C m^−2^ d^−1^ (Eq. 6).

6 $$GPP \,\left(\mathrm{kg \,C \,}{\mathrm{m}}^{-2} \,{\mathrm{d}}^{-1}\right)=\frac{GPP*0.001}{8}$$

### Fire severity assessment

#### Differenced normalized burn rate (ΔNBR)

Burned area severity or ΔNBR can be defined as the difference between the pre-fire NBR and the post-fire NBR. A high ΔNBR value indicates severe fire damage and a negative ΔNBR indicates a high rate of vegetation growth after the burn has occurred.

Burn severity was mapped using the NBR-based bi-temporal index (ΔNBR). Fire severity metrics are based on the normalized burning rate (NBR, Eq. 7)^80^, which include the delta normalized burn rate (ΔNBR, Eq. 8)^81^. The values can be obtained by applying the shortwave (SWIR) and near-infrared (NIR) lengths, as areas damaged by fires usually have high reflectance values in the SWIR range and low values in the NIR range. Conversely, healthy vegetation is identified with high reflectance values in the NIR range and low values in the SWIR range^82^, since the NIR band is sensitive to the chlorophyll content in vegetation and the SIWR band is suitable for identifying moisture content in vegetation and soil^83^. Consequently, the differentiation between burning areas and healthy vegetation can be determined by the high values in the NIR or SWIR spectral regions^84^

7 $$NBR= \frac{(NIR-SWIR)}{(NIR+SWIR)}$$

8 $$\Delta NBR={NBR}_{PRE\_FIRE}-{NBR}_{POST\_FIRE}$$

The process of classifying the severity of vegetation fires using the ΔNBR index was carried out on a pixel-by-pixel basis using MODIS/MOD13Q1 images. The images were acquired in the period before and after the fire season (May to November) in the Pantanal during the time series from 2001 to 2022, using the Google Earth Engine platform. The ΔNBR index class values according to^85^ where a high ΔNBR value indicates severe burning and negative values indicate a high rate of re-sprouting and growth after fires (Table 3), according to^85^.

**Table 3:** Classification of the ΔNBR index using Google Earth Engine.

	Severity level	ΔNBR range (scale for 10^3^)	ΔNBR range (No scale)
	Enhanced regrowth high (ERH)	− 500 to − 251	− 0.500 to 0.251
	Enhanced regrowth low (ERL)	− 250 to − 101	− 0.100 to − 1.101
	Unburned (UN)	− 100 to + 99	− 0.100 to + 0.99
	Low severity (LS)	+ 100 to + 269	+ 0.100 to 0.269
	Moderate-low severity (MLS)	+ 270 to + 439	+ 0.270 to + 0.439
	Moderate-high severity (MHS)	+ 440 to + 659	+ 0.400 to + 0.659
	High severity (HS)	+ 660 to + 1300	+ 0.660 to + 1.300

### Determining risk areas

In order to determine the risk areas, which are areas with a higher probability of environmental fires, the MLS, MSH and HS classes of the ∆NBR classification were separated for each year of the time series, and then the average was made for the study period, indicating the frequent fire sites in the Pantanal biome.

Initially, the boxplot graph was constructed to show the behavior of the ΔNBR index classes evaluated over the time series using the GGPLOT2 package^86^, on the R software. Subsequently, Mann–Kendall's test at 5% probability level was used to identify trends over the time series (2001–2022) for each variable (Gross Primary Production, precipitation, and CO_2_ flux) and the ΔNBR classes^87,88^. Finally, the data was submitted to the Pettitt test^89^ at 5% probability, which identifies the point at which there is a sudden change in the mean of a time series. For both tests, the analyses were carried out using the Rbio software^90^.

Pearson's correlations (r) between fire foci, rainfall, GPP, and CO_2_ flux were estimated and represented by a correlation and scatter plot. The analyses were carried out using the Rbio^90^ and R using the ggplot2 package. Afterward, data were subjected to principal component analysis (PCA) to assess the relationship between the variables, locations, and the years of study, as well as between the severity classes of the ΔNBR index and the years of study.

PCA is a multivariate statistical analysis that converts an original dataset (X1, X2, …, Xp) into another same-sized dataset (Y1, Y2, …, Yp), reducing the data volume with minimal loss of information. Principal components come from the linear combination of the original variables, which are independent of each other, retaining a maximum of information^31^.

### Supplementary Information

Supplementary Tables.

## Data Availability

The datasets used and/or analysed during the current study available from the corresponding author on reasonable request.
