# Supplementary material for: Dynamics of major environmental disasters involving fire in the Brazilian Pantanal
Source: Sci Rep. 2023 Dec 7;13:21669. doi: 10.1038/s41598-023-49154-6 (PMC10709611; doi:10.1038/s41598-023-49154-6)
Supplement: Supplementary file 1 — Supplementary Tables. [file 41598_2023_49154_MOESM1_ESM.docx]

**Supplementary Material**

|  | | | | | | | | | | |
| --- | --- | --- | --- | --- | --- | --- | --- | --- | --- | --- |
| **Mato Grosso** | | | | | | **Mato Grosso Do Sul** | | | | |
| **Years** | **GPP** | **Rain** | **CO_2_**  **Flux** | **Fire foci** | **Density** | **GPP** | **Rain** | **CO_2_**  **Flux** | **Fire foci** | **Density** |
| 2001 | 0.01 | 1295.89 | 0.89 | 3136 | 0.0006 | 0.01 | 1240.15 | 1.05 | 5786 | 0.0006 |
| 2002 | 0.01 | 1055.59 | 0.85 | 5515 | 0.0010 | 0.01 | 799.43 | 1.19 | 20243 | 0.0021 |
| 2003 | 0.01 | 1229.23 | 0.86 | 2079 | 0.0004 | 0.01 | 1216.56 | 0.95 | 4401 | 0.0005 |
| 2004 | 0.01 | 1199.53 | 0.83 | 5280 | 0.0010 | 0.01 | 1113.10 | 0.82 | 9357 | 0.0010 |
| 2005 | 0.01 | 1141.67 | 0.91 | 6156 | 0.0012 | 0.01 | 1103.00 | 1.17 | 18400 | 0.0019 |
| 2006 | 0.02 | 1408.13 | 0.98 | 1081 | 0.0002 | 0.01 | 1229.33 | 1.03 | 4668 | 0.0005 |
| 2007 | 0.01 | 1196.22 | 0.92 | 4601 | 0.0009 | 0.01 | 1134.06 | 1.21 | 13779 | 0.0014 |
| 2008 | 0.02 | 1195.54 | 0.85 | 3725 | 0.0007 | 0.01 | 1137.06 | 0.89 | 5456 | 0.0006 |
| 2009 | 0.02 | 1245.04 | 0.92 | 935 | 0.0002 | 0.02 | 1069.04 | 0.90 | 10657 | 0.0011 |
| 2010 | 0.02 | 924.48 | 1.04 | 7506 | 0.0014 | 0.02 | 904.65 | 1.16 | 8408 | 0.0009 |
| 2011 | 0.02 | 1244.52 | 0.92 | 3963 | 0.0007 | 0.02 | 1217.83 | 0.99 | 4412 | 0.0005 |
| 2012 | 0.02 | 1197.13 | 0.41 | 2289 | 0.0004 | 0.02 | 1084.94 | 0.69 | 11329 | 0.0012 |
| 2013 | 0.02 | 1078.96 | 0.54 | 3199 | 0.0006 | 0.02 | 1145.52 | 0.58 | 4345 | 0.0004 |
| 2014 | 0.02 | 1505.54 | 0.52 | 759 | 0.0001 | 0.02 | 1385.18 | 0.60 | 1988 | 0.0002 |
| 2015 | 0.02 | 1224.02 | 0.22 | 2469 | 0.0005 | 0.02 | 1203.73 | 0.26 | 5901 | 0.0006 |
| 2016 | 0.02 | 1290.85 | 0.57 | 1360 | 0.0003 | 0.02 | 1279.80 | 0.77 | 9118 | 0.0009 |
| 2017 | 0.03 | 1337.85 | 0.54 | 3261 | 0.0006 | 0.02 | 1349.52 | 0.54 | 7094 | 0.0007 |
| 2018 | 0.03 | 1513.32 | 0.57 | 919 | 0.0002 | 0.03 | 1365.38 | 0.52 | 2098 | 0.0002 |
| 2019 | 0.03 | 950.74 | 0.49 | 3529 | 0.0007 | 0.02 | 906.90 | 0.61 | 18637 | 0.0019 |
| 2020 | 0.02 | 762.85 | 0.82 | 31112 | 0.0058 | 0.02 | 902.40 | 0.74 | 19047 | 0.0020 |
| 2021 | 0.02 | 1105.40 | 0.67 | 5246 | 0.0010 | 0.02 | 990.49 | 0.93 | 14049 | 0.0014 |
| 2022 | 0.03 | 984.98 | 0.46 | 465 | 0.0001 | 0.02 | 861.80 | 0.61 | 2369 | 0.0002 |

**Supplementary Table 1** Values of fire foci, rain, CO_2_ flux, fire foci density, and GPP distributed within the Pantanal of MT and MS between the years 2001 to 2022.

| **Years** | **Mean** | **Standard Error** | **Median** | **Standard Deviation** | **Sample Variance** | **Minimum** | **Maximum** | **Sum** |
| --- | --- | --- | --- | --- | --- | --- | --- | --- |
| **2001** | 740.92 | 322.16 | 146.5 | 1115.99 | 1245442.81 | 35 | 3075 | 8891 |
| **2002** | 2150.58 | 760.23 | 743 | 2633.53 | 6935463.54 | 27 | 6055 | 25807 |
| **2003** | 546.42 | 175.39 | 307.5 | 607.57 | 369141.36 | 64 | 2051 | 6557 |
| **2004** | 1211.83 | 544.56 | 378 | 1886.41 | 3558556.88 | 38 | 6376 | 14542 |
| **2005** | 2072.50 | 1027.51 | 633.5 | 3559.40 | 12669321.55 | 29 | 12182 | 24870 |
| **2006** | 492.58 | 169.32 | 196 | 586.53 | 344011.90 | 35 | 1749 | 5911 |
| **2007** | 1535.17 | 865.32 | 272 | 2997.56 | 8985366.70 | 10 | 10422 | 18422 |
| **2008** | 765.17 | 295.99 | 212 | 1025.33 | 1051295.79 | 34 | 3141 | 9182 |
| **2009** | 1013.25 | 220.05 | 887.5 | 762.28 | 581065.66 | 107 | 2353 | 12159 |
| **2010** | 1322.83 | 515.20 | 615 | 1784.69 | 3185133.42 | 51 | 6083 | 15874 |
| **2011** | 699.58 | 242.87 | 229.5 | 841.33 | 707842.45 | 4 | 2237 | 8395 |
| **2012** | 1141.33 | 466.54 | 339 | 1616.12 | 2611859.15 | 171 | 4827 | 13696 |
| **2013** | 628.75 | 215.86 | 226 | 747.75 | 559137.11 | 30 | 2494 | 7545 |
| **2014** | 221.83 | 76.13 | 120 | 263.73 | 69555.42 | 21 | 886 | 2662 |
| **2015** | 697.42 | 214.22 | 300 | 742.07 | 550661.17 | 59 | 2143 | 8369 |
| **2016** | 875.17 | 363.09 | 197 | 1257.77 | 1581992.52 | 47 | 4039 | 10502 |
| **2017** | 863.33 | 390.89 | 303.5 | 1354.07 | 1833516.06 | 49 | 4811 | 10360 |
| **2018** | 252.58 | 108.94 | 62.5 | 377.39 | 142423.36 | 12 | 1330 | 3031 |
| **2019** | 1848.25 | 621.91 | 706 | 2154.35 | 4641226.93 | 142 | 5837 | 22179 |
| **2020** | 4106.08 | 1638.69 | 1260 | 5676.59 | 32223649.36 | 289 | 18137 | 49273 |
| **2021** | 1599.92 | 701.45 | 299 | 2429.91 | 5904445.36 | 99 | 6624 | 19199 |
| **2022** | 337.08 | 71.33 | 297 | 247.09 | 61054.45 | 0 | 719 | 4045 |

**Supplementary Table 2**- Descriptive statistics of fire foci, via the FIRMS platform, detected in the Brazilian Pantanal biome between 2001 and 2022.

| **Years** | **Mean** | | **Standard Error** | | **Median** | | **Standard Deviation** | | | **Sample Variance** | | **Minimum** | | **Maximum** | | **Sum** |
| --- | --- | --- | --- | --- | --- | --- | --- | --- | --- | --- | --- | --- | --- | --- | --- | --- |
| **2001** | | 740.92 | | 322.16 | | 146.5 | | 1115.99 | 1245442.81 | | 35 | | 3075 | | 8891 | |
| **2002** | | 154.59 | | 33.30 | | 127.96 | | 115.35 | 13306.65 | | 19.38 | | 388.55 | | 1855.02 | |
| **2003** | | 203.82 | | 44.78 | | 201.01 | | 155.14 | 24068.22 | | 21.18 | | 518.71 | | 2445.79 | |
| **2004** | | 192.72 | | 36.87 | | 195.46 | | 127.73 | 16315.94 | | 13.95 | | 406.64 | | 2312.63 | |
| **2005** | | 187.06 | | 44.75 | | 174.09 | | 155.01 | 24027.85 | | 13.23 | | 544.40 | | 2244.67 | |
| **2006** | | 219.79 | | 46.17 | | 254.37 | | 159.93 | 25577.90 | | 19.24 | | 451.06 | | 2637.46 | |
| **2007** | | 194.19 | | 52.42 | | 136.77 | | 181.60 | 32979.08 | | 9.76 | | 570.71 | | 2330.28 | |
| **2008** | | 194.38 | | 48.44 | | 186.26 | | 167.80 | 28155.42 | | 14.87 | | 577.86 | | 2332.60 | |
| **2009** | | 192.84 | | 39.14 | | 154.03 | | 135.57 | 18379.93 | | 45.96 | | 404.57 | | 2314.08 | |
| **2010** | | 152.43 | | 34.36 | | 163.55 | | 119.04 | 14170.78 | | 9.37 | | 365.91 | | 1829.13 | |
| **2011** | | 205.20 | | 56.53 | | 175.74 | | 195.83 | 38349.42 | | 19.50 | | 526.24 | | 2462.35 | |
| **2012** | | 190.17 | | 33.32 | | 199.82 | | 115.41 | 13319.22 | | 8.09 | | 353.50 | | 2282.07 | |
| **2013** | | 185.37 | | 36.97 | | 163.87 | | 128.07 | 16400.96 | | 15.92 | | 413.23 | | 2224.48 | |
| **2014** | | 240.89 | | 43.96 | | 234.09 | | 152.29 | 23191.92 | | 17.08 | | 449.94 | | 2890.72 | |
| **2015** | | 202.31 | | 32.30 | | 215.82 | | 111.91 | 12523.21 | | 22.98 | | 356.56 | | 2427.75 | |
| **2016** | | 214.22 | | 44.69 | | 189.00 | | 154.80 | 23962.07 | | 17.63 | | 544.68 | | 2570.65 | |
| **2017** | | 223.95 | | 47.15 | | 223.96 | | 163.34 | 26681.40 | | 14.53 | | 452.52 | | 2687.37 | |
| **2018** | | 239.89 | | 55.51 | | 172.96 | | 192.29 | 36976.41 | | 23.46 | | 551.68 | | 2878.70 | |
| **2019** | | 154.80 | | 31.78 | | 156.19 | | 110.07 | 12116.21 | | 24.29 | | 299.32 | | 1857.63 | |
| **2020** | | 138.77 | | 31.82 | | 131.43 | | 110.23 | 12149.70 | | 16.18 | | 319.95 | | 1665.25 | |
| **2021** | | 174.66 | | 46.12 | | 122.14 | | 159.77 | 25527.69 | | 7.04 | | 478.83 | | 2095.89 | |
| **2022** | | 153.90 | | 30.78 | | 133.21 | | 106.62 | 11368.56 | | 14.65 | | 360.37 | | 1846.78 | |

**Supplementary Table 3** -Descriptive statistics of precipitation in mm, via CHIRPS data, in the Brazilian Pantanal biome between 2001 and 2022.

| **Years** | **Mean** | **Standard Error** | **Median** | **Standard Deviation** | **Sample Variance** | **Minimum** | **Maximum** | **Sum** |
| --- | --- | --- | --- | --- | --- | --- | --- | --- |
| **2001** | 1.93 | 0.30 | 1.94 | 1.03 | 1.07 | 0.55 | 3.79 | 23.22 |
| **2002** | 2.03 | 0.32 | 1.94 | 1.10 | 1.22 | 0.47 | 3.40 | 24.39 |
| **2003** | 1.81 | 0.33 | 1.73 | 1.16 | 1.34 | 0.33 | 3.69 | 21.76 |
| **2004** | 1.65 | 0.34 | 1.67 | 1.17 | 1.38 | 0.43 | 4.42 | 19.76 |
| **2005** | 2.08 | 0.42 | 1.91 | 1.46 | 2.13 | 0.41 | 4.75 | 24.96 |
| **2006** | 2.01 | 0.39 | 1.66 | 1.35 | 1.81 | 0.52 | 5.10 | 24.06 |
| **2007** | 2.14 | 0.50 | 1.91 | 1.72 | 2.96 | 0.34 | 6.33 | 25.64 |
| **2008** | 1.74 | 0.30 | 1.80 | 1.05 | 1.11 | 0.25 | 3.51 | 20.94 |
| **2009** | 1.82 | 0.29 | 1.87 | 1.01 | 1.01 | 0.17 | 3.24 | 21.83 |
| **2010** | 2.20 | 0.43 | 2.17 | 1.50 | 2.25 | 0.19 | 4.90 | 26.39 |
| **2011** | 1.91 | 0.38 | 1.97 | 1.30 | 1.69 | 0.11 | 4.82 | 22.89 |
| **2012** | 1.10 | 0.38 | 0.64 | 1.31 | 1.72 | -0.33 | 4.07 | 13.18 |
| **2013** | 1.12 | 0.29 | 1.29 | 1.01 | 1.02 | -0.29 | 2.78 | 13.47 |
| **2014** | 1.12 | 0.37 | 0.96 | 1.30 | 1.68 | -0.26 | 4.31 | 13.48 |
| **2015** | 0.48 | 0.21 | 0.32 | 0.72 | 0.52 | -0.48 | 1.71 | 5.76 |
| **2016** | 1.34 | 0.36 | 1.22 | 1.26 | 1.59 | -0.33 | 4.07 | 16.08 |
| **2017** | 1.08 | 0.29 | 1.09 | 1.00 | 1.01 | -0.32 | 2.70 | 13.00 |
| **2018** | 1.08 | 0.20 | 1.01 | 0.69 | 0.48 | -0.17 | 2.25 | 12.99 |
| **2019** | 1.10 | 0.34 | 0.68 | 1.17 | 1.37 | -0.42 | 3.03 | 13.21 |
| **2020** | 1.56 | 0.51 | 0.83 | 1.77 | 3.13 | -0.24 | 4.92 | 18.67 |
| **2021** | 1.60 | 0.52 | 1.37 | 1.79 | 3.22 | -0.55 | 4.54 | 19.19 |
| **2022** | 1.07 | 0.34 | 0.69 | 1.18 | 1.40 | -0.26 | 3.40 | 12.87 |

**Supplementary Table 4 -** Descriptive Statistics CO_2_ Flux values via MODIS /MODO9A1, detected in the Brazilian Pantanal biome, between 2001 and 2022.

| **Years** | **Mean** | **Standard Error** | **Median** | **Standard Deviation** | **Sample Variance** | **Minimum** | **Maximum** | **Sum** |
| --- | --- | --- | --- | --- | --- | --- | --- | --- |
| **2001** | 1.93 | 0.30 | 1.94 | 1.03 | 1.07 | 0.55 | 3.79 | 23.22 |
| **2002** | 2.03 | 0.32 | 1.94 | 1.10 | 1.22 | 0.47 | 3.40 | 24.39 |
| **2003** | 1.81 | 0.33 | 1.73 | 1.16 | 1.34 | 0.33 | 3.69 | 21.76 |
| **2004** | 1.65 | 0.34 | 1.67 | 1.17 | 1.38 | 0.43 | 4.42 | 19.76 |
| **2005** | 2.08 | 0.42 | 1.91 | 1.46 | 2.13 | 0.41 | 4.75 | 24.96 |
| **2006** | 2.01 | 0.39 | 1.66 | 1.35 | 1.81 | 0.52 | 5.10 | 24.06 |
| **2007** | 2.14 | 0.50 | 1.91 | 1.72 | 2.96 | 0.34 | 6.33 | 25.64 |
| **2008** | 1.74 | 0.30 | 1.80 | 1.05 | 1.11 | 0.25 | 3.51 | 20.94 |
| **2009** | 1.82 | 0.29 | 1.87 | 1.01 | 1.01 | 0.17 | 3.24 | 21.83 |
| **2010** | 2.20 | 0.43 | 2.17 | 1.50 | 2.25 | 0.19 | 4.90 | 26.39 |
| **2011** | 1.91 | 0.38 | 1.97 | 1.30 | 1.69 | 0.11 | 4.82 | 22.89 |
| **2012** | 1.10 | 0.38 | 0.64 | 1.31 | 1.72 | -0.33 | 4.07 | 13.18 |
| **2013** | 1.12 | 0.29 | 1.29 | 1.01 | 1.02 | -0.29 | 2.78 | 13.47 |
| **2014** | 1.12 | 0.37 | 0.96 | 1.30 | 1.68 | -0.26 | 4.31 | 13.48 |
| **2015** | 0.48 | 0.21 | 0.32 | 0.72 | 0.52 | -0.48 | 1.71 | 5.76 |
| **2016** | 1.34 | 0.36 | 1.22 | 1.26 | 1.59 | -0.33 | 4.07 | 16.08 |
| **2017** | 1.08 | 0.29 | 1.09 | 1.00 | 1.01 | -0.32 | 2.70 | 13.00 |
| **2018** | 1.08 | 0.20 | 1.01 | 0.69 | 0.48 | -0.17 | 2.25 | 12.99 |
| **2019** | 1.10 | 0.34 | 0.68 | 1.17 | 1.37 | -0.42 | 3.03 | 13.21 |
| **2020** | 1.56 | 0.51 | 0.83 | 1.77 | 3.13 | -0.24 | 4.92 | 18.67 |
| **2021** | 1.60 | 0.52 | 1.37 | 1.79 | 3.22 | -0.55 | 4.54 | 19.19 |
| **2022** | 1.07 | 0.34 | 0.69 | 1.18 | 1.40 | -0.26 | 3.40 | 12.87 |

**Supplementary Table 5**- Descriptive statistics of Gross Primary Production via MODES/MOD17A2 data, detected in the Brazilian Pantanal biome.

| **Areas classified with ∆NBR in the Pantanal biome** | | | | | | | |
| --- | --- | --- | --- | --- | --- | --- | --- |
| **Years** | **E R H** | **E R L** | **UN** | **L S** | **MlS** | **MHS** | **HS** |
| 2001 | 220365 | 1637737 | 8374838 | 3624125 | 876682 | 231304 | 42237 |
| 2002 | 58719 | 382038 | 4288511 | 5860873 | 3262071 | 981094 | 150689 |
| 2003 | 108558 | 836937 | 7654664 | 5098639 | 1111418 | 158465 | 11758 |
| 2004 | 90402 | 866691 | 7784442 | 4940834 | 1136218 | 159930 | 14063 |
| 2005 | 408279 | 2104452 | 8280813 | 3423556 | 671301 | 94062 | 11150 |
| 2006 | 356143 | 1346292 | 7766858 | 4370218 | 944409 | 163743 | 19138 |
| 2007 | 264837 | 1555245 | 6938906 | 4503018 | 1365504 | 303934 | 60558 |
| 2008 | 138274 | 1012140 | 7475193 | 4760241 | 1323105 | 248227 | 34316 |
| 2009 | 351078 | 1535262 | 7903290 | 3968445 | 973829 | 193771 | 22171 |
| 2010 | 49400 | 445999 | 6214564 | 5784318 | 1929734 | 487229 | 81016 |
| 2011 | 74290 | 417963 | 5674804 | 5912993 | 2160888 | 628864 | 125183 |
| 2012 | 68762 | 548154 | 7097109 | 5052084 | 1736746 | 446117 | 49228 |
| 2013 | 64992 | 419453 | 6121582 | 6110695 | 1908054 | 321365 | 46080 |
| 2014 | 53319 | 237318 | 5659854 | 5953088 | 2331788 | 652260 | 90601 |
| 2015 | 133264 | 892345 | 8017750 | 4655098 | 1081661 | 197624 | 17985 |
| 2016 | 104591 | 383534 | 5130548 | 5891117 | 2543914 | 760159 | 157880 |
| 2017 | 71839 | 583401 | 7407346 | 5142997 | 1420665 | 306654 | 36420 |
| 2018 | 411044 | 2225963 | 8310917 | 3210495 | 694556 | 129790 | 16269 |
| 2019 | 31757 | 160943 | 3525425 | 5948604 | 3467453 | 1384794 | 455989 |
| 2020 | 124381 | 649596 | 3157148 | 4889479 | 3545719 | 1821386 | 811559 |
| 2021 | 189414 | 921295 | 5401455 | 5475483 | 2046096 | 730934 | 243093 |
| 2022 | 514515 | 2036587 | 8611002 | 3230203 | 487693 | 93596 | 7371 |

**Supplementary Table 6**– Classification of the Brazilian Pantanal territory using the ∆NBR index, using MODIS/MOD13Q1, between the years 2001 to 2022.
